# Predicting length of stay ranges by using novel deep neural networks

**DOI:** 10.1016/j.heliyon.2023.e13573

**Published:** 2023-02-09

**Authors:** Hong Zou, Wei Yang, Meng Wang, Qiao Zhu, Hongyin Liang, Hong Wu, Lijun Tang

**Affiliations:** a Department of General Surgery, The General Hospital of Western Theater Command (Chengdu Military General Hospital), Chengdu, 610083, China; b Department of Liver Surgery & Liver Transplantation, State Key Laboratory of Biotherapy and Cancer Center, West China Hospital, Sichuan University and Collaborative Innovation Center of Biotherapy, Chengdu 610044, Sichuan Province, China; c Department of Urology, The General Hospital of Western Theater Command (Chengdu Military General Hospital), Chengdu, 610083, China; d Department of Traditional Chinese Medicine, The General Hospital of Western Theater Command (Chengdu Military General Hospital), Chengdu, 610083, China; e Department of Obstetrics and Gynecology, The General Hospital of Western Theater Command (Chengdu Military General Hospital), Chengdu, 610083, China

**Keywords:** Length of stay, Prediction, MIMIC-III, Deep learning, Accuracy

## Abstract

**Background and aims:**

Accurately predicting length of stay (LOS) is considered a challenging task for health care systems globally. In previous studies on LOS range prediction, researchers commonly pre-classified the LOS ranges, which were the same for all patients in the same classification, and then utilized a classifier for prediction. In this study, we innovatively aimed to predict the specific LOS range for each patient (the LOS range was different for each patient).

**Methods:**

In the modified deep neural network (DNN), the overall sample error (root mean square error (RMSE) method), the estimated sample error (ERR_pred_ method), the probability distribution with different loss functions (Dis_pred__Loss1, Dis_pred__Loss2, and Dis_pred__Loss3 method), and the generative adversarial networks (WGAN-GP for LOS method) are used for LOS range prediction. The Medical Information Mart for Intensive Care III (MIMIC-III) database is used to validate these methods.

**Results:**

The RMSE method is convenient for LOS range prediction, but the predicted ranges are all consistent in the same batch of samples. The ERR_pred_ method can achieve better prediction results in samples with low errors. However, the prediction effect is worse in samples with larger errors. The Dis_pred__Loss1 method encounters a training instability problem. The Dis_pred__Loss2 and Dis_pred__Loss3 methods perform well in making predictions. Although WGAN-GP for LOS method does not show a substantial advantage over other methods, this method might have the potential to improve the predictive performance.

**Conclusion:**

The results show that it is possible to achieve an acceptable accurate LOS range prediction through a reasonable model design, which may help physicians in the clinic.

## 1 Introduction

Accurate prediction of length of stay (LOS) is deemed a challenging task for health care systems globally [1]. On the one hand, predicting LOS is crucial for hospital management and bed capacity planning and thus affects the access, quality, and efficiency of health care services [2]. On the other hand, predicting LOS can effectively help clinicians estimate the severity of a patient's condition and determine medical treatment [3].

There have been several studies on predicting LOS [4]. The methods used in these studies included human prediction by experienced physicians [5], prediction by using regression models [[6], [7], [8]], prediction by using machine learning models [[9], [10], [11], [12], [13], [14]], and prediction by using deep learning models [1,[15], [16], [17], [18], [19], [20], [21]].

Although researchers have used different prediction methods, these studies can still be broadly classified into two types of tasks according to the results of prediction [4,22,23]: (1) Estimating the precise value of LOS (the results of these predictions are numerical). This type of task is also known as a regression task. (2) Pre-classification based on LOS (binary classification or multiclassification) and then utilizing a classifier for prediction. This type of task is also known as a classification task. In studies of classification tasks, the 5 days [24], 7 days [[25], [26], [27]], and the 3rd quartile of LOS [21,28] are the most regularly used dichotomous time intervals. The LOS ranges were the same for all patients in the same classification, as they were preset. The patients whose LOS was around the cutoff point may be classified into different classifications, although their actual difference is not significant. Predicting the LOS range for each patient individually in accordance with his or her clinical characteristics is probably more valuable.

However, to the best of our knowledge, there are no relevant studies using deep learning models to predict the LOS ranges of each individual patient. In this study, through model and algorithm improvements, we innovatively attempted to predict the specific LOS range for each patient (the LOS range was different for each patient).

## 2 Methods

### 2.1 Data collection and preprocessing

The Medical Information Mart for Intensive Care III (MIMIC-III) database, which includes clinical data related to more than 60,000 unidentified patients in the ICU at Beth Israel Deaconess Medical Center from 2001 to 2012, is a publicly available intensive care database maintained by the Massachusetts Institute of Technology (MIT) Laboratory of Computational Physiology [29]. The database contains virtually all electronic patient record data that can be collected, including demographics, vital signs, test results, exam findings, operations, and medication use. The MIMIC-III database can be used for analytical studies, including epidemiology, clinical decision planning, and electronic tool development [16,30,31].

After a localized deployment of the MIMIC-III v1.4 database, the PostgreSQL database management system v14.2 software (The PostgreSQL Global Development Group & Regents of the University of California) was used to manage and extract data. The extracted data included features consisting of patient demographics, diagnoses, vital signs, test results, treatments, other relevant information, and LOS labels. Relevant measures, including fluid balance and severity assessment, were also constructed based on official MIMIC database documentation (https://github.com/MIT-LCP/mimic-code/tree/main/mimic-iii) [32].

Data from 38,597 patients (>15 years) were included in the study. Feature variables are partially missing. Missing discrete variable data are represented by N/A, and missing continuous variables are filled by their mean values.

A 9:1 ratio is used to divide the training set (34,737) and test set (3,860). According to hadm_id (hospitalization number) in the MIMIC-III database, 3 datasets with different feature sizes and LOS labels are created. Dataset A contains 27 basic characteristic variables, admission time, and discharge time. There are fewer features, but the features are explicitly linearly related to the label in dataset A. Dataset B contains 27 basic characteristic variables, admission time, and no discharge time. There are also fewer features, and the features are not explicitly linearly related to the label in dataset B. Dataset C contains 136 basic characteristic variables, admission time, and without discharge time, referring to a benchmarking study on the MIMIC-III database [16]. There are more features, although they do not have an explicit linear relationship related to the label in dataset C.

### 2.2 Prediction methods

#### 2.2.1 Predicting the LOS ranges by using the overall sample error

In this method, a standard deep neural network (DNN) is used for LOS range prediction. After the training in the training set, the predicted LOS (LOS_pred_) and the root mean square error (RMSE) are obtained. Then, [LOS_pred_-α × RMSE, LOS_pred_+α × RMSE] is used as an estimate of the LOS range. This method is called the RMSE method in this study.

#### 2.2.2 Predicting the LOS range by using an estimated sample error

**Fig. 1:**
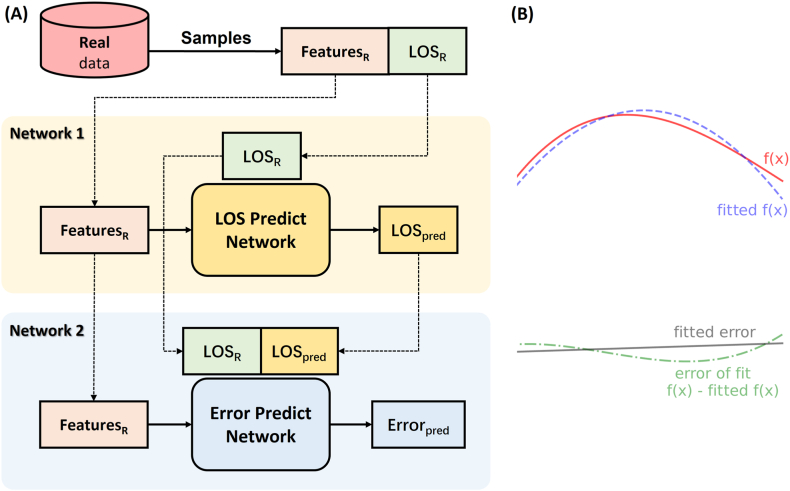
**Model of networks for length of stay and error prediction.** A. Network1 is a standard deep neural network structure with features as input and the length of stay (LOS) as output, while Network2 has features as input and (LOSR-LOSpred) as output. B. Given that x is the set of features, and the features and LOSR correspond to f(x) (red line), then the role of Network1 is equivalent to fitting f(x) (fitted f(x), purple line). By calculating the difference between LOSR and LOSpred, the error between the real value and the predicted value of a sample can be determined (f(x)-fitted f(x), green line). The role of Network2 is equivalent to fitting this error (fitted error, black line). . (For interpretation of the references to colour in this figure legend, the reader is referred to the Web version of this article.)

After using the DNN for LOS prediction, the obtained LOS_pred_ and real LOS (LOS_R_) are substituted into another DNN again (as shown in Fig. 1A). The first DNN corresponds to the fitting of the correspondence between features and LOS_R_. The second DNN corresponds to the fitting of the correspondence between features and errors (LOS_R_-LOS_pred_) (as shown in Fig. 1B). With such an improved DNN structure, two prediction values, namely, LOS_pred_ and the predicted error (ERR_pred_), can be obtained. Then, [LOS_pred_-α × ERR_pred_, LOS_pred_+α × ERR_pred_] is used as an estimate of the LOS range. This method is called the ERR_pred_ method in this study.

#### 2.2.3 Predicting the LOS range by using the probability distribution

It is assumed that the LOS of a specific patient is a probability distribution conforming to a normal distribution, and this patient's LOS_R_ is μ_0_. Then, this LOS distribution is consistent with $N{(\mu 0,\sigma 0}^{2})$, σ0→0. If the output of the standard DNN is modified to 2, and by reasonable adjustment, it is possible to make output1→μ0 and output2→σ0, output1 is referred to as μ_pred_, and output2 is referred to as σ_pred_. In this way, we can obtain the predicted probability distribution of a sample $N\left({\mu \text{pred},\sigma \text{pred}}^{2}\right)$. Next, we estimate the LOS range by using [μ_pred_-α × σ_pred_, μ_pred_+α × σ_pred_].

In this study, we use 3 different loss functions for LOS range prediction. The derivation process of the loss function is detailed in Appendix 1.

##### 2.2.3.1 Dis_pred__Loss1 method

$$\text{Loss}\;\text{function}\;1=\frac{e^{-z}\;\mathrm{erf}\left(0.707\sqrt{e^{-2z}}\left(\mu \text{pred}-\mu 0+1\right)\right){-e}^{-z}\;\mathrm{erf}\left(0.707\sqrt{e^{-2z}}\left(\mu \text{pred}-\mu 0-1\right)\right)}{2\sqrt{e^{-2z}}}$$

(1) $$\mathrm{erf}\left(x\right)=\frac{2}{\sqrt{\pi }}\int_{0}^{x}e^{{-\eta }^{2}}d\eta {\sigma \text{pred}=e}^{z}$$

**Fig. 2:**
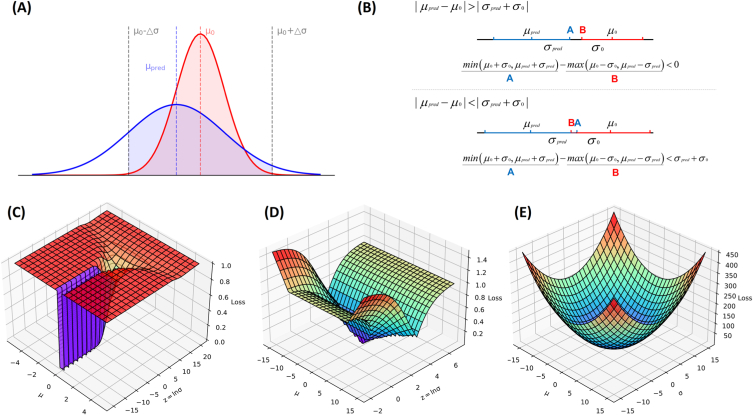
**Predicting the LOS range by using the probability distribution.** A. The Dispred_Loss1 method approximates the difference between the true probability (red shaded area) and the predicted probability (blue shaded area) in the range of μ0±Δμ (Δμ>0). B. The Dispred_Loss2 method approximates the overlap range of the intervals [μ0-σ0, μ0+σ0] and [μpred-σpred, μpred+σpred]. C. Visualization of the loss function in the Dispred_Loss1 method. D. Visualization of the loss function in the Dispred_Loss2 method. E. Visualization of the loss function in the Dispred_Loss3 method. . (For interpretation of the references to colour in this figure legend, the reader is referred to the Web version of this article.)

Loss function of Dispred_Loss1 method is given as Equation (1). The Dispred_Loss1 method utilizes the probability density function and the probability distribution function. This method approximates the difference between the true probability and the predicted probability in the range of μ_0_±Δμ (Δμ>0) (as shown in Fig. 2A).

As shown in Fig. 2C, when μpred→μ0 and z→−∞, Lossfunction1→0.

##### 2.2.3.2 Dis_pred__Loss2 method

(2) $$\text{Loss}\;\text{function}\;2=1-\frac{{\min(\mu 0+e}^{z0}{,\mu \text{pred}+e}^{\text{zpred}}{)-\max(\mu 0-e}^{z0}{,\mu \text{pred}-e}^{\text{zpred}})}{2\left(\mu 0+\mu \text{pred}\right)}{\sigma \text{pred}=e}^{z}$$

Loss function of Dispred_Loss1 method is given as Equation (2). The Dis_pred__Loss2 method approximates the overlap range of the intervals [μ_0_-σ_0_, μ_0_+σ_0_] and [μ_pred_-σ_pred_, μ_pred_+σ_pred_] (as shown in Fig. 2B).

As shown in Fig. 2D, when μpred→μ0 and z→z0, Lossfunction2→0

##### 2.2.3.3 Dis_pred__Loss3 method

(3) $${\text{Loss}\;\text{function}\;3=\left(\mu \text{pred}-\mu 0\right)}^{2}{+\left(\sigma \text{pred}-\sigma 0\right)}^{2}+\lambda \frac{{\left(\mu \text{pred}-\mu 0\right)}^{2}}{{\sigma \text{pred}}^{2}+\varepsilon }\left(\varepsilon =0.000001\right)$$

Loss function of Dispred_Loss1 method is given as Equation (3). The Dis_pred__Loss3 method approximates the Wasserstein distance between the real distribution and the predicted distribution and adds a correlation penalty term of μ and σ. The Wasserstein distance is a fine method for measuring the distance of two distributions with gradient smoothing.

As shown in Fig. 2E, when μpred→μ0 and σpred→σ0, Lossfunction3→0

#### 2.2.4 Predicting the LOS range by using generative adversarial networks

**Fig. 3:**
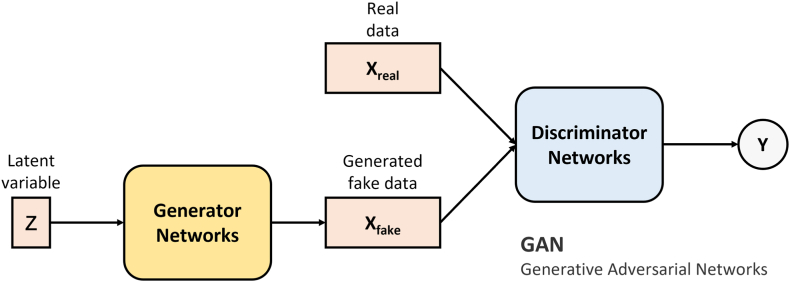
**Basic generative adversarial networks.** The generator generates fake data from a random variable Z. The discriminator distinguishes between fake data and real data. In constant iterations, the generator can generate fake data that even the discriminator cannot distinguish from the real data.

The generative adversarial network (GAN) is an excellent generative model in deep learning (the basic GAN structure is shown in Fig. 3) and one of the most popular research directions in artificial intelligence [33]. At present, there are more than 500 improved GAN variants, which have shown unexpectedly good results in data enhancement, image and medical image conversion, electronic health record data generation, biomedical data generation, and data interpolation [[34], [35], [36]].

**Fig. 4:**
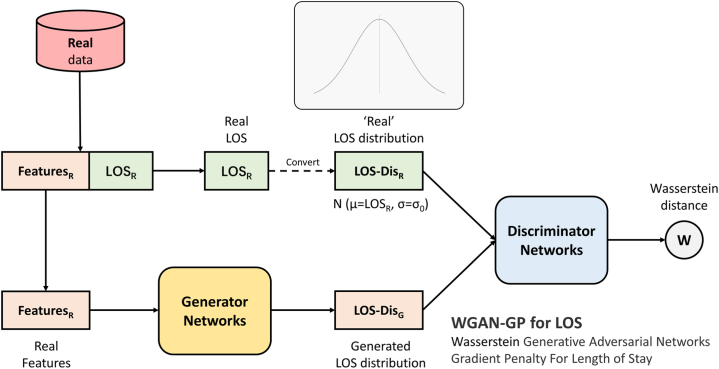
**Wasserstein GAN with a gradient penalty for LOS.** Features generate a predicted sample distribution through the generator. Then, the ‘real’ LOS distribution and the generated LOS distribution are determined by the discriminator. In this improved GAN, the discriminator approximates the fit to the Wasserstein distance with the addition of a penalty term on the gradient.

In this study, we improve the basic GAN for LOS range prediction; this improved GAN is called the Wasserstein GAN with a gradient penalty [37] for the LOS (WGAN-GP for LOS) (as shown in Fig. 4). It is also assumed that LOS conforms to a distribution. Features generate a predicted sample distribution through the generator. Then, the real distribution and the generated distribution are determined by the discriminator. After repeated adversarial training, the generator in WGAN-GP for LOS can generate realistic LOS distributions.

It is possible to generate a distribution with an arbitrary number by using the generator in the trained WGAN-GP for LOS. In this study, the number of samples generated by the generator is 500. The mean (mean_pred_) and standard deviation (STD_pred_) of these 500 samples are calculated. Then, we estimate the LOS range by using [mean_pred_-α × STD_pred_, mean_pred_+α × STD_pred_]. In this study, this method is called WGAN-GP for LOS method.

### 2.3 Determination of the LOS ranges and measure

In this study, we attempted to predict the specific LOS range for each patient. The predicted outcomes were generally expressed as [lower limit of LOS, upper limit of LOS]. The lower and upper limits of LOS were estimated by LOS±α × error. In different methods, the predicted LOS value and error value differed. We summarized the determination of the LOS ranges in different methods in Table 1. The change in the α value led to a change in the LOS range and reflected the change in the overall error of the prediction sample.

**Table 1:** Determination of the LOS ranges in different methods.

Methods	Determination
RMSE1	LOS_pred_^2^ ± α × RMSE
ERR_pred_^3^	LOS_pred_ ± α × ERRpred
Dis_pred__Loss1^4^	*μ*_pred_ ± α × σpred
Dis_pred__Loss2^5^	*μ*_pred_ ± α × σpred
Dis_pred__Loss3^6^	*μ*_pred_ ± α × σpred
WGAN-GP for LOS^7^	mean_pred_ ± α × STDpred^8^

Description: ^1^ RMSE, root mean square error; ^2^ LOSpred, predicted length of stay; ^3^ ERRpred, predicted error; ^4–6^ Dispred_Loss1-3, prediction of the LOS range using the probability distribution with loss function 1–3; ^7^ WGAN-GP for LOS, wasserstein generative adversarial network with a gradient penalty for the length of stay; ^8^ STDpred, predicted standard deviation.

Accuracy was the major metric for evaluation we adopted. The prediction was considered correct when the LOS_R_ was within the predicted LOS range. The accuracy for the same overall prediction error (48 and 96) in different methods was evaluated. The overall prediction error in different methods with sufficient accuracy (>95%) was also evaluated. The RMSE method is considered the benchmark method because it is easy to calculate the overall prediction error.

### 2.4 Ethical issues

The study was approved by the Institutional Review Board of the General Hospital of Western Theater Command. Because the study does not affect clinical treatment and care and all protected health information is deidentified, the requirement for individual patient consent is waived.

### 2.5 Experimental environment and statistical analysis

This study was conducted on a computer with an NVIDIA(R) RTX(R) 2060 GPU and Intel(R) Xeon(R) CPU E−2224G processor. PostgreSQL database management system v14.1 (The PostgreSQL Global Development Group & Regents of the University of California, USA) and Python 4.1.0 (Python Software Foundation, Wilmington, DE, USA) were used for data extraction and preprocessing, model development and validation, visualization and statistical analysis. The DNN and WGAN-GP for LOS were implemented through PyTorch packages in Python. Descriptive statistics are used to describe patient characteristics and are expressed as the mean (STD), median [quartiles], or absolute numbers (proportions) as appropriate. P < 0.05 was considered statistically significant.

## 3 Results

### 3.1 Validation of the training set and test set

**Fig. 5:**
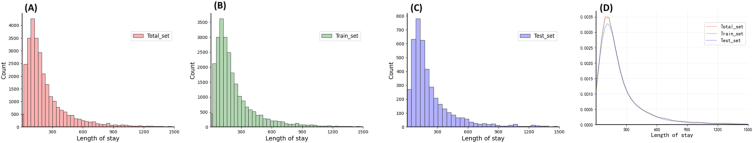
**The LOS in the training set and the test set.** A. Histogram of LOS distribution in the total set. B. Histogram of LOS distribution in the training set. C. Histogram of LOS distribution on the test set. D. Kernel density map of LOS distribution in different datasets. LOS is expressed in hours. Values above 1500 are not included in the figure.

The LOSs in the total set, training set, and test set were 6.75 [3.88–12.07], 6.77 [3.88–12.07], and 6.59 [3.86–11.87] days, respectively (as shown in Fig. 5(A-D)), with no significant differences between groups (P > 0.05). This result is consistent with previous reports.

### 3.2 Predicting the LOS range

#### 3.2.1 Using an overall sample error

**Fig. 6:**
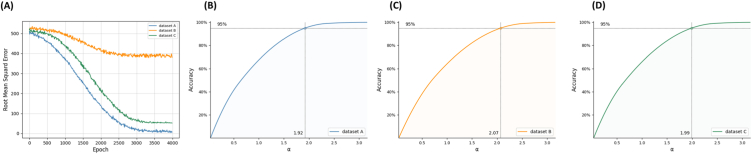
**Predicting the LOS range by using an overall sample error.** A. Variation in the RMSE method when training on different datasets. B. Accuracy of LOS prediction at different LOS ranges in dataset A. C. Accuracy of LOS prediction at different LOS ranges in dataset B. D. Accuracy of LOS prediction at different LOS ranges in dataset C.

In this method, the DNN is used directly for LOS prediction with data from datasets A, B, and C. After the training model reaches convergence, the RMSE is shown in Fig. 6A. As expected, on dataset A, the trained model obtained the smallest RMSE (15.56) due to a clear linear relationship between LOS and features. The largest RMSE (380.54) is obtained on dataset B with fewer features. A moderate RMSE (52.05) is obtained on dataset C with more features.

The LOS range is estimated by using the RMSE method (LOS_pred_±α × RMSE), and the results are shown in Fig. 6B, C, and 6D. In dataset A, when α is 1.92, an accuracy of 95% can be obtained. In datasets B and C, this α value is 2.07 and 1.99, respectively.

#### 3.2.2 Using an estimated sample error

**Fig. 7:**
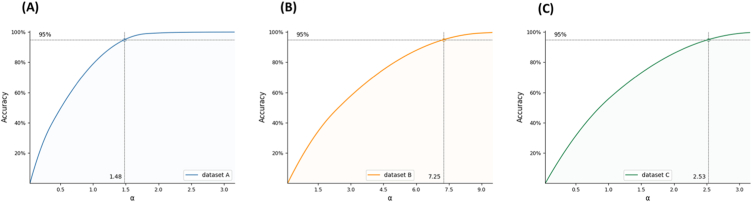
**Predicting the LOS range by using an estimated sample error.** A. Accuracy of LOS prediction at different LOS ranges in dataset A. B. Accuracy of LOS prediction at different LOS ranges in dataset B. C. Accuracy of LOS prediction at different LOS ranges in dataset C.

The results of LOS range prediction by using the ERR_pred_ method are shown in Fig. 7(A-C). The ERR_pred_ method performs well on dataset A. A 95% accuracy can be obtained at α = 1.48, which is lower than the value obtained by using the RMSE method directly to perform the prediction. However, as the overall sample error increases, the performance of this method becomes more inefficient. A 95% accuracy can be obtained at α = 2.53 on dataset C and α = 7.25 on dataset B.

#### 3.2.3 Using the probability distribution

In this part of the study, we investigate the effects of 3 different loss functions. The μ_pred_ values predicted by the Dis_pred__Loss1, Dis_pred__Loss2, and Dis_pred__Loss3 methods are 158.64 ± 44.54, 156.24 ± 41.85, and 158.49 ± 44.76 h, respectively. There is no significant difference in the μ_pred_ of these three methods (P > 0.05). However, the σ_pred_ values predicted by these methods were 60.52 ± 35.71, 31.12 ± 17.84, and 41.94 ± 28.94 h, with considerable differences between the groups (P < 0.05).

**Fig. 8:**
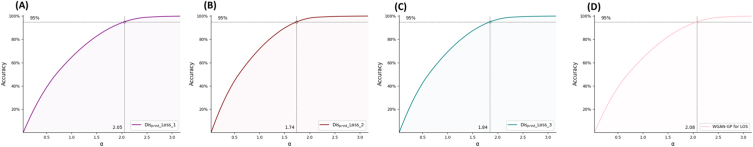
**Predicting the LOS range by using the probability distribution and GAN.** A. Accuracy of LOS prediction at different LOS ranges by using the Dispred_Loss1 method. B. Accuracy of LOS prediction at different LOS ranges by using the Dispred_Loss2 method. C. Accuracy of LOS prediction at different LOS ranges by using the Dispred_Loss3 method. D. Accuracy of LOS prediction at different LOS ranges by using WGAN-GP for LOS method.

The results of LOS range prediction by using the probability distribution are shown in Fig. 8A, B, and 8C. A 95% accuracy can be obtained at α = 2.05 by using the Dis_pred__Loss1 method, at α = 1.74 by using the Dis_pred__Loss2 method, and at α = 1.84 by using the Dis_pred__Loss3 method.

#### 3.2.4 Using GAN

The mean value of the overall samples generated by the generator does not differ significantly from LOS_R_ (236.52 ± 192.89 vs. 246.36 ± 204.35, P > 0.05). The result of the LOS range prediction by using WGAN-GP for LOS method is shown in Fig. 8D. A 95% accuracy can be obtained at α = 2.08.

### 3.3 Comparison of the prediction performance

**Fig. 9:**
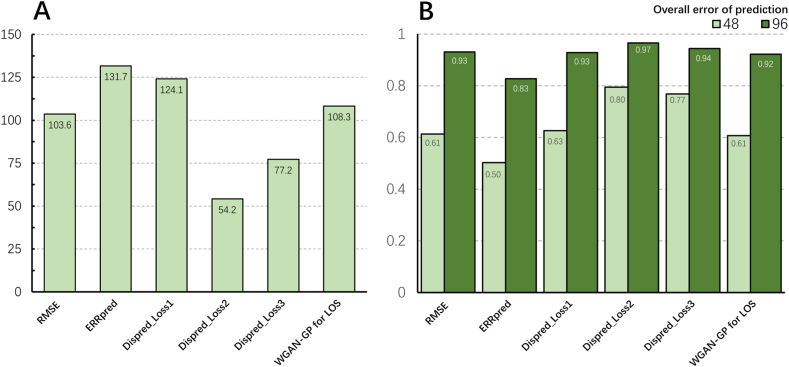
**Comparison of prediction performance.** A. The overall prediction error with sufficient accuracy (>95%) in different methods. B. The accuracy for the same overall prediction error (48 and 96) in different methods.

Based on these results, we compared the prediction performance of various methods on dataset C, which was more compatible with clinical practice. As shown in Fig. 9A, the overall prediction error obtained by the RMSE method was 103.6 when sufficient prediction accuracy (>95%) was achieved. The overall prediction error obtained by the ERR_pred_ (131.7), Dis_pred__Loss1 (124.1), and WGAN-GP for LOS (108.3) method were higher, and the overall prediction error obtained by the Dis_pred__Loss2 (54.2) and Dis_pred__Loss3 (77.1) methods was lower.

As shown in Fig. 9B, when the overall prediction error was consistently set to 48, the prediction accuracy of the RMSE method was 61.3%. The ERR_pred_ method (50.2%) had the lowest prediction accuracy, and the Dis_pred__Loss2 method (79.5%) had the highest prediction accuracy. When the overall prediction error was set to 96, the prediction accuracy of the RMSE method was 93.1%. Except for the ERR_pred_ method (82.7%), all methods achieved a favorable prediction accuracy (>90%). Of these, the Dis_pred__Loss2 method (96.6%) had the highest prediction accuracy.

## 4 Discussion

In addition to being a major indicator of the consumption of hospital resources, LOS can be considered a metric that can be used to identify the severity of illness [21] and provide an enhanced understanding of the flow of patients through hospital care units and environments; understanding the flow of patients is important in the evaluating the operational functions of various care systems [38].

In previous studies, most of the prediction indicators are the precise values of LOS; estimating these values is usually considered a regression task. Obtaining the smallest error between the predicted LOS and the actual LOS is the objective of regression tasks. In clinical practice, a precise estimate of LOS may not be particularly valuable. Intuitively, assuming that the LOS of two patients is precisely 200 h and 202 h, clinicians may classify them as having similar levels of severity, and thus, devote similar medical resources to them. However, assuming that the LOS of two patients is imprecise, which are [200−240] hours and [100−140] hours, clinicians may be likely to classify them as having different levels of severity and devote different medical resources to them.

Recently, several studies on predicting LOS range have been conducted. In these studies, the predicting LOS is successfully transformed from a regression task into a classification task, resulting in promising predicted outcomes. The LOS ranges are the same for all patients in the same classification as they are preset. Predicting the respective LOS range for each patient may provide greater assistance to clinicians in making medical decisions.

Additionally, accurate estimates of LOS for individual samples may be more valuable. It is assumed that the overall estimation error is acceptable in a batch of samples. Of these, 90% of the estimations have a small error, and the other 10% have a large error. When we evaluate a specific patient, we must consider the possibility that this estimation is in the range of estimations with a large error if we do not know whether the estimation is part of the 90% or the 10%; this lack of knowledge directly affects our determination of the confidence of the prediction results. Furthermore, when the model for predicting LOS range performs well (with sufficiently high accuracy), we can infer not only the length of in-hospital stay by the value of LOS but also the predicted effect by the range of LOS.

The first method we adopt is to use the DNN directly and to predict the LOS range by using the LOS_pred_ and RMSE (LOS_pred_±α × RMSE). This method is relatively simple and successfully converts the regression task into a binary classification task. Our results show that an accuracy of 95% can be obtained on datasets with different overall errors when α is approximately 2. This method achieves the aim of estimating the precision and accuracy of the sample simultaneously. However, the error range is consistent for all samples in this method, and it is unclear which samples have larger errors and which ones have smaller errors.

We attempt to estimate the error again for a single sample by using another DNN. In this way, we obtain the predicted values LOS_pred_ and Error_pred_ and estimate the LOS range by using LOS_pred_±α × Error_pred_. Obtaining the overall estimation error by using the ERR_pred_ method is the same as obtaining the overall estimation error by using the RMSE method because the same DNN is used to estimate the LOS_pred_. The ERR_pred_ method performs well on dataset A, where the overall sample error is low. By this method, 95% accuracy is obtained at approximately α = 1.5, which is lower than the direct estimation obtained by using the RMSE method. However, the performance of this method becomes considerably worse as the overall sample error increases. On dataset C with a medium overall sample error, 95% accuracy is obtained at approximately α = 2.5. On dataset B with a larger overall sample error, 95% accuracy is obtained at α > 7 probably because there is still an error in the error estimation [39]. When the overall sample error increases, the systematic error in the error estimation is substantially magnified and becomes unacceptable.

We further improve the DNN model. An important assumption we make is to convert the real LOS value into distribution ${N(\mu 0,\sigma 0}^{2})$. The real LOS is equivalent to this distribution when LOSR=μ0 and σ0→0. Then, we use the DNN to predict this distribution by changing the model and setting different loss functions so that we can obtain a predicted distribution ${N(\mu \text{pred},\sigma \text{pred}}^{2})$. We can estimate the LOS range by using μ_pred_±α × σ_pred_. Based on this assumption, we validate dataset C, which is more inclined to the actual data.

We obtain better results by using the Dis_pred__Loss1 method compared to using the ERR_pred_ method. However, as shown in Fig. 3C, the loss function has little gradient variation over most of the range, thus inducing instability [40] and gradient disappearance during training. The gradient clipping technique partly ameliorated the training instability. However, this method still has difficult convergence and a long training time, which is similar to the problem encountered in the training of some GAN models [41].

The Dis_pred__Loss2 method and Dis_pred__Loss3 method demonstrate favorable results, and the mean values of σ are only slightly larger than the RMSE of the DNN, indicating that neither model greatly increased the overall error. The Dis_pred__Loss2 method also encounters gradient disappearance when σ is too large. Therefore, the gradient clipping technique is also used in this method. In the Dis_pred__Loss3 method, the model is fitted too quickly, making σ converge to 0 if the two-dimensional Wasserstein distance is used directly. Adding the correlation penalty term for μ and σ ($\lambda \frac{{\left(\mu \text{pred}-\mu 0\right)}^{2}}{{\sigma \text{pred}}^{2}+\varepsilon }$) solves this problem considerably. ε is added (ε = 0.000001) to avoid gradient explosion due to σ converging to 0. λ is a hyperparameter that regulates the degree of convergence of the distribution tendency μ or σ. Additionally, our results show that the variance of σ obtained using the Dis_pred__Loss3 method is larger than that obtained using the Dis_pred__Loss2 method, thus possibly indicating that Dis_pred__Loss3 is more accurate in predicting some results and less accurate in predicting others. We might estimate the prediction confidence level by varying σ. The two methods may be useful in different situations.

GAN is an excellent generative model in deep learning and one of the most popular research directions in artificial intelligence [33]. GAN's inspiring ideas on adversarial learning have penetrated deeply into various aspects of deep learning, giving rise to a range of new research directions and various applications. We also use a modified GAN in this study to predict the LOS range [37]. It is possible to generate a distribution of samples directly by using WGAN-GP for LOS method. This distribution can be any distribution (normal, skewed, uniform, or other). As with other GAN models, WGAN-GP for LOS method greatly increases the computational effort and the training time. Although WGAN-GP for LOS method does not currently show a substantial advantage over other models, we believe that with further improvements to GAN, this method may be one of the ways to further improve prediction performance.

The number of clinical data, especially with accurate labels and rare diseases and conditions, is very limited for the following reasons: (1) the diagnostic and patient labeling process depends highly dependent on experienced human experts and is often very time-consuming [42]; (2) obtaining detailed results of laboratory tests and other medical features, while becoming more feasible than ever before using modern facilities, is still very expensive [43]; (3) it is difficult to correlate medical data collected by different health information systems due to obstacles between systems, resulting in fewer medical data available for scientific research [44]; and (4) privacy concerns and regulations make it more complicated to collect and secure enough medical data with the detailed information needed [45]. These challenges, which are distinct in health care, prevent current machine learning or deep learning models from using sufficient available and high-quality labeled data to their advantage.

Our results preliminarily showed that it is feasible to achieve better predictions with relatively limited medical data by improving the model and the algorithm. The RMSE method successfully transformed the prediction of the LOS value into the LOS range. We compared the RMSE method with other methods as a benchmark method in this study. The Dispred_Loss2 and Dispred_Loss3 methods achieved better prediction accuracy than the RMSE method with the same overall error. Additionally, compared to the RMSE method, the Dispred_Loss2 and Dispred_Loss3 methods also reduced the overall prediction error with sufficient accuracy (>95%). Although WGAN-GP for LOS method does not currently show a substantial advantage over other models, we believe that with further improvements to GAN, this method may be one of the ways to further improve the prediction performance.

This study still has a few limitations. First, only data from the mimic database are used for training and validation. These results have not been confirmed in the external training set or in clinical practice. Second, for the interpretability of the results, we adopt only a simple DNN model and do not use some complex models, such as convolutional neural networks (CNNs), recurrent neural networks (RNNs), and long short-term memory (LSTM), which have the potential to further improve prediction [[46], [47], [48]]. Third, in this study, our prediction range uses LOS_pred_±α × value_pred_ (although in different forms). Prediction accuracy is also achieved by adjusting α values in various models. Directly using [upper limit, lower limit] to achieve the most accurate prediction does not arise as a research objective in this study. However, this may be a more important research objective, even if this objective is more difficult and may make the model more complicated. Based on the results of other studies on GANs, WGAN-GP for LOS method may have greater potential in this respect.

In summary, the results of this study show that it is possible to achieve an acceptable LOS range estimate through a reasonable model design, which helps clinicians determine the severity of the patient's condition. In the near future, artificial intelligence may become a trusted solution to assist in medical decision-making.

## Author contribution statement

Hong Zou, Wei Yang, Meng Wang, Qiao Zhu, Hongyin Liang, Hong Wu, Lijun Tang: Conceived and designed the experiments; Performed the experiments; Analyzed and interpreted the data; Contributed reagents, materials, analysis tools or data; Wrote the paper.

## Funding statement

Lijun Tang was supported by National Key Clinical Specialty Army Construction Project [41732113], Sichuan Province Science and Technology Support Program [2019YJ0277].

## Data availability statement

Data will be made available on request.

## Declaration of interest's statement

The authors declare no competing interests.
