# Supplementary material for: Predicting length of stay ranges by using novel deep neural networks
Source: Heliyon. 2023 Feb 9;9(2):e13573. doi: 10.1016/j.heliyon.2023.e13573 (PMC9958433; doi:10.1016/j.heliyon.2023.e13573)
Supplement: Multimedia component 1 [file mmc1.docx]

Appendix 1

1. Loss function in the Dispred_Loss1 method

- In the training set, the length of stay of the sample is assumed to conform to a normal distribution, .
- Its probability density function:

(1)

- Its probability distribution function:

$\text{Φ}(x)=\int f(x)dx$ (2)

- Assuming that △μ is an error that we can accept, the probability that the sample fits within the range of (μ0-△μ, μ0+△μ) is

$\text{Φ}'(x)=\int_{\mu0-\triangle\mu}^{\mu0+\triangle\mu} f(x)dx$ (3)

- obviously, for this sample

$$\text{Φ}'(x|\triangle\mu=1)\to1$$

- Assume that our predicted length of stay is also normally distributed and that its distribution is consistent with
- Its probability density function:

(4)

- The probability that the distribution is within the range of (μ0-△μ, μ0+△μ | △μ= 1) is

(5)

- if and only if and ,
- Therefore, we can set the loss function as

(6)

(7)

(8)

- Since μ > 0, we can artificially set $\mu=e^{z}$, thus ensuring that μ > 0
- In this case

$Loss\approx1-\int_{x-1}^{x+1} \frac{1}{\sqrt{2\pi}\text{e}^{\text{z}}}e^{-\frac{(x-\mu pred)^{2}}{2\text{e}^{2\text{z}}}}$ (9)

- The definite integral can be solved for

$Loss\approx\frac{e^{-z}erf\left(0.707\sqrt{e^{-2z}}\left(x-\mu+1\right)\right)-e^{-z}erf\left(0.707\sqrt{e^{-2z}}\left(x-\mu-1\right)\right)}{2\sqrt{e^{-2z}}}$ (10)

$erf(x)=\frac{2}{\sqrt{\pi}}\int_{0}^{x} e^{-\eta^{2}}\text{d}\eta$ (11)

- if and $\text{z}\to\text{-}\infty$, $Loss\to0$

2. Loss function in the Dispred_Loss2 method

- The length of stay of the sample is also assumed to conform to a normal distribution,
- Our objective is to make the two distributions as similar in shape as possible, whereby our objective is and
- We can consider the objective as making the range (μ_0_-σ_0_, μ_0_+σ_0_) intersect with another range (μ_pred_-σ_pred_, μ_pred_+σ_pred_) as large as possible

(12)

- When and ,

($Value<1$)

When and ,

($Value<1$)

When ,

($Value<1$)

When ,

($Value<0$)

If and , The function can obtain the maximum value 1.

- Since μ > 0, we can artificially set $\mu=e^{z}$, thus ensuring that μ > 0

(13)

- Then the loss function:

$$Loss=1-Value$$

(14)

- if and , $Loss\to0$

3. Loss function in the Dispred_Loss3 method

- Measurement tools to measure the difference between the two distributions include KL scatter, JS scatter and Wasserstein distance. The Wasserstein distance has a smoother gradient. Therefore, in some cases training the model using Wasserstein distance is better than KL and JS scatter.
- Wasserstein distance:

(15)

- when P1~ *N*(μ_0_，σ_0_^2^)，P2~ *N*(μ_pred_，σ_pred_^2^)

(16)

- If and ,

$$W(P1,P2)=0$$

- The loss function is constructed by adding correlation penalty term of μ and σ to the Wasserstein distance:

$(\varepsilon=0.000001)$ (17)

λ is a hyperparameter that regulates the degree of convergence of the distribution tendency μ or σ
